# Dr. Hope on the Treatment of Acute Rheumatism

**Published:** 1842-04-23

**Authors:** 


*Dr. Hope on the treatment of Acute Rheumatism.*—After one full bleeding, or even two in robust subjects, but without any bleeding in the feeble and delicate, I give, every night, gr. vii. of calomel with one and a half of opium, or gr. x. of calomel with gr. ij. of opium, according to the age and severity of the symptoms. This is followed every morning by inf. sennæ c. ℥iss. magnesiæ sulph. ʒij., and mannæ ʒj., which should act at least four or five times. In addition, I generally give the following draught thrice a day, as it has appeared to me to expedite the cure—partly, perhaps, by the additional opiate, and partly by the sedative effect of the colchicum. ℞ Vini colchici, m. xv. ad xx.; pulv. ipecac. comp. gr. v.; mist. salin. ʒx.; syrupi, ʒj ♏ ft. haustus.

When the pain and swelling are greatly abated if not almost gone, (which often happens within two days, and almost always within four,) I omit the calomel, or, if the gums become in the slightest degree tender, I omit it even earlier. The opium, I continue, to the extent of gr. j. or iss. at bedtime, and in severe cases I add a grain at noon—for, without an anodyne, the pains are apt to recur. I also continue the colchicum draughts and the senna draught.

No local treatment is necessary beyond warm or cold applications according as the patient finds them agreeable.

If the patient is not well in a week, I consider it a case of exception; and the exceptions are generally in those who are subject to rheumatism, and who, therefore, usually have it in a more obstinate, chronic form. The advantages of this plan are, 1. The patient is generally well, sound, and fit for work in a week or ten days after the pains have ceased. 2. The gums are rarely affected—especially if it be previously ascertained that the patient has not a peculiar susceptibility of the action of mercury. 3. *It is rare to see inflammation of the heart supervene*, if the treatment is early commenced: I think that about one case in twelve would be the maximum in my practice. 4. If the slightest symptoms of pericarditis or endocarditis do supervene, a few additional doses of calomel and opium, (as gr. v. of calomel with gr. j. of opium, every four or six hours,) will generally affect the constitution in twenty or thirty hours, which, with two or three cuppings or leechings on the præcordial region, almost always places the patient in a state of safety. I have never lost a patient by rheumatic inflammation of the heart since I have employed this plan, and I have been told by other hospital physicians that they have been scarcely less successful.

